# First WHO Meeting of Stakeholders on Elimination of Gambiense Human African Trypanosomiasis

**DOI:** 10.1371/journal.pntd.0003244

**Published:** 2014-10-23

**Authors:** Peter Holmes

**Affiliations:** University of Glasgow, Glasgow, Scotland, United Kingdom; Institute of Tropical Medicine, Belgium

Human African Trypanosomiasis (HAT), also known as Sleeping Sickness, has been one of the most important human diseases in Africa because of widespread epidemics in the past, its very high level of mortality, and its negative influence on the development of rural populations. Two forms of the disease exist, one chronic form in West and Central Africa caused by *Trypanosoma brucei gambiense* (>95% of current cases), and an acute form in East and Southern Africa caused by *Trypanosoma brucei rhodesiense* (<5% of current cases).

During the 1960s HAT was brought under control by the colonial health systems, but unfortunately the rarity of HAT cases led to a decline in awareness of how the disease could return and subsequently, to a lack of interest in disease surveillance and a decrease in disease control. With this decrease in control and surveillance activities, the disease re-emerged, reaching epidemic proportions by the end of the 20th century, with the majority of human infections caused by *T. b. gambiense*. The alarming rise in the number of cases stimulated international efforts to reverse the epidemiological trend and reduce the incidence of the disease, using enhanced surveillance and improved access to diagnosis and treatment in endemic countries [1], [2]. These activities have been complemented by improvements in the epidemiological knowledge of the disease and the production of highly detailed disease distribution maps of affected countries [3].

As a result of these outstanding efforts by national sleeping sickness programmes and the support of international organizations led by the World Health Organization (WHO), and involving key pharmaceutical companies and major international donors, remarkable results have been achieved during the past 15 years, with the number of new reported cases falling to 6,228 in 2013 [4]. This has involved strong collaboration and coordination of all these stakeholders and the maintenance of a permanent and open dialogue.

Based on the advances achieved in the control of the disease, in 2012 the WHO Strategic and Technical Advisory Group for Neglected Tropical Diseases made the decision to target elimination of gambiense HAT as a public health problem by 2020 and to target zero incidence of the disease by 2030. The 2020 target was included in the WHO roadmap for elimination and control of neglected tropical diseases [5], and it is defined as the reduction of gambiense HAT incidence to less than 1 new case per 10,000 population at risk, in at least 90% of foci with fewer than 2000 cases reported globally [4]–[6].

In 2013 this elimination target was endorsed by the disease endemic countries [7]–[8], the WHO Expert Committee on Control and Surveillance of HAT [6], and the London Declaration on Neglected Tropical Diseases [9]. More recently it was adopted by the 66th World Health Assembly in the resolution WHA66.12 [10].

In order to move forward towards gambiense HAT elimination, the first WHO meeting of stakeholders on elimination of gambiense HAT was held in Geneva (Switzerland) from 25 to 27 March 2014. Participants came from national sleeping sickness control programmes, groups developing new tools to fight HAT, international and non-governmental organizations involved in HAT control and major international donors including private sector companies Sanofi and Bayer Healthcare, bilateral agencies like the Belgian Development Cooperation, and philanthropic organizations such as the Bill and Melinda Gates Foundation and the Wellcome Trust.

The meeting reviewed the current epidemiological status of the disease and the recent achievements and challenges for moving towards the gambiense HAT elimination goal. The stakeholders also analysed the current status of important technical aspects of HAT control that are currently in development and will greatly assist in bringing about the elimination of HAT. These include the development of new drugs currently in clinical trials and new diagnostics tools that are becoming available, research on some unresolved epidemiological aspects such as the potential role of asymptomatic carriers and animal reservoirs and recently improved methods of vector control. The meeting also examined the establishment of mechanisms for monitoring and evaluation of the elimination process as well as the confirmation of outcomes. Finally, the ways and mechanisms through which collaboration and coordination among stakeholders can be strengthened and organized were considered. As the number of cases of HAT continue to decrease it will be critical that the international stakeholders maintain their support, that HAT surveillance, treatment, and control activities are integrated within health services, and that the national ownership of the control programmes is achieved in order to bring about the sustainable elimination of the disease and to avoid the repetition of the painful experience of the last century.

The first WHO meeting of stakeholders on elimination of gambiense HAT held in Geneva, Switzerland in March 2014 concluded by issuing a declaration for the elimination of gambiense HAT, which appeals to the international community at large and to disease-endemic countries for their commitment, political support, and essential resources to achieve the elimination goal and establishes a network under the leadership of WHO to ensure coordinated, strengthened, and sustained efforts to eliminate the disease [11]. The declaration of the first stakeholders meeting on gambiense HAT elimination can be viewed at: http://www.who.int/trypanosomiasis_african/meeting_declaration_2014/en/.
